# Supplementary material for: MetaRibo-Seq measures translation in microbiomes
Source: Nat Commun. 2020 Jun 29;11:3268. doi: 10.1038/s41467-020-17081-z (PMC7324362; doi:10.1038/s41467-020-17081-z)
Supplement: Supplementary file 10 — Supplementary Data 7 [file 41467_2020_17081_MOESM10_ESM.zip › File2/Confidence_High_Taxonomy/345872_out.krona.html]

Javascript must be enabled to view this page.

members
magnitude
magnitudeUnassigned
count
unassigned
taxon
rank

345872\_out

27

superkingdom
2
27


SRS142890\_contig\_number\_8048
1
27
1224
phylum

1236
26
class

order
26
91347

543
26
family
3

SRS063127\_contig\_number\_contig-100\_2667.2668SRS077127\_contig\_number\_8250SRS147151\_contig\_number\_22509


SRS140492\_contig\_number\_27537
1
23
561
genus

species

SRS012273\_contig\_number\_45070SRS017191\_contig\_number\_contig-100\_988.121377SRS017521\_contig\_number\_48071SRS018313\_contig\_number\_9206SRS019601\_contig\_number\_13181SRS046502\_contig\_number\_2084SRS049402\_contig\_number\_14269SRS064276\_contig\_number\_1595SRS075947\_contig\_number\_19544SRS077646\_contig\_number\_182SRS098881\_contig\_number\_10998SRS105082\_contig\_number\_17690SRS142781\_contig\_number\_10965SRS143014\_contig\_number\_contig-100\_127.128SRS143423\_contig\_number\_28516SRS143596\_contig\_number\_contig-100\_241.242SRS144219\_contig\_number\_contig-100\_367.368SRS144362\_contig\_number\_7879SRS146888\_contig\_number\_contig-100\_77.99198SRS146996\_contig\_number\_4403SRS147022\_contig\_number\_14006SRS148196\_contig\_number\_contig-100\_6006.118431
22
562
